# Myostain is involved in ginsenoside Rb1-mediated anti-obesity

**DOI:** 10.1080/13880209.2022.2074056

**Published:** 2022-05-31

**Authors:** Hong-Shi Li, Jiang-Ying Kuang, Gui-Jun Liu, Wei-Jie Wu, Xian-Lun Yin, Hao-Dong Li, Lei Wang, Tao Qin, Wen-Cheng Zhang, Yuan-Yuan Sun

**Affiliations:** a The Key Laboratory of Cardiovascular Remodeling and Function Research, Chinese Ministry of Education, Chinese National Health Commission and Chinese Academy of Medical Sciences, The State and Shandong Province Joint Key Laboratory of Translational Cardiovascular Medicine, Department of Cardiology, Qilu Hospital, Cheeloo College of Medicine, Chinese Ministry of Education, Shandong University, Jinan, China; b Department of Cardiology, The Second Hospital, Cheeloo College of Medicine, Shandong University, Jinan, China; c Grade 2018, School of Basic Medical Sciences, Clinical Medicine (5 + 3), Cheeloo College of Medicine, Shandong University, Jinan, China; d Department of Emergency Surgery, Qilu Hospital, Cheeloo College of Medicine, Shandong University, Jinan, China

**Keywords:** Obesity, MSTN, FNDC5

## Abstract

**Context:**

Obesity, one of the major public health problems worldwide, has attracted increasing attention. Ginsenoside Rb1 is the most abundant active component of *Panax ginseng* C.A.Mey (Araliaceae) and is reported to have beneficial effects on obesity and diabetes. However, the mechanisms by which Rb1 regulates obesity remain to be explored.

**Objective:**

This paper intends to further explore the mechanism of Rb1 in regulating obesity.

**Materials and methods:**

The C57BL/6 obese mice were divided into two groups: the control (CTR) and Rb1. The CTR group [intraperitoneally (ip) administered with saline] and the Rb1 group (ip administered with Rb1, 40 mg/kg/d) were treated daily for four weeks. *In vitro*, Rb1 (0, 10, 20, 40 μM) was added to differentiated C2C12 cells and Rb1 (0, 20, 40 μM) was added to 3T3-L1 cells. After 24 h, total RNA and protein from C2C12 cells and 3T3-L1 cells were used to detect myostatin (MSTN) and fibronectin type III domain-containing 5 (FNDC5) expression.

**Results:**

Rb1 reduced the body weight and adipocyte size. Improved glucose tolerance and increased basic metabolic activity were also found in Rb1 treated mice. MSTN was downregulated in differentiated C2C12 cells, 3T3-L1 cells and adipose tissues upon Rb1 treatment. FNDC5 was increased after Rb1 treatment. However, MSTN overexpression attenuated Rb1-mediated decrease accumulation of lipid droplets in differentiated 3T3-L1 adipocytes.

**Discussion & Conclusions:**

Rb1 may ameliorate obesity in part through the MSTN/FNDC5 signalling pathway. Our results showed that Rb1 can be used as an effective drug in the treatment of human obesity.

## Introduction

Obesity, as one of the main public health problems worldwide, can lead to dyslipidemia, insulin resistance, type 2 diabetes, hypertension, heart failure, tumours, and obstructive sleep apnoea. The main characteristic of obesity is the large accumulation of triglycerides (TG) in adipose tissue, which is due to adipocyte hyperplasia (increased number) or hypertrophy (increased size) or even both. It was believed that adipocyte hypertrophy occurred before adipocyte hyperplasia and was the major mechanism of fat mass expansion (Faust et al. 1978; Duncan et al. 2007). Adipose tissue is mainly composed of fat cells, including white fat, brown fat and beige fat. When the body's energy intake far exceeds the amount consumed, the excess part will be stored as white fat. The treatment of obesity is an effective way to prevent a variety of diseases.

*Ginseng*, a traditional Chinese medicine, has been harvested and used for thousands of years in Eastern Asia. It has been revealed that Rb1, the most abundant bioactive component of *Panax ginseng* C.A.Mey (Araliaceae), can improve leptin sensitivity (Wu et al. 2018), ameliorate glycolipid metabolism (Shang et al. 2008), reduce triglyceride accumulation (Park et al. 2008) and reduce fatty liver (Shen et al. 2013). Although aquaporin 7 (AQP7) (Guo et al. 2020), adenosine 5′-monophosphate (AMP)-activated protein kinase (AMPK) (Shen et al. 2013), phosphatidylinositol 3-kinase (PI3K)/Akt signalling pathway (Chen et al. 2019) and peroxisome proliferator-activated receptor γ (PPARγ) (Song et al. 2020) have been reported to be included in the effects of Rb1 on adipocytes and adipose tissue, the mechanisms of Rb1 in regulating body weight are still need to be explored.

Myostatin (MSTN; also called GDF8) is a member of the transforming growth factor β (TGFβ) superfamily. It is predominantly secreted by skeletal muscle. High expression of MSTN can inhibit muscle growth and development (McPherron et al. 1997). Studies have shown that there was increased muscle mass, reduced fat deposition, improved insulin sensitivity, enhanced fatty acid oxidation, and promoted resistance to obesity in MSTN knock out mice (Bernardo et al. 2010; Lebrasseur 2012; Zhang et al. 2012). It was found that there is higher expression of MSTN in adult skeletal muscle and lower expression levels in adult adipose tissue. Type 2 diabetes mellitus patients showed significantly lower MSTN levels and higher irisin levels than controls (García-Fontana et al. 2016). Deletion of MSTN prevents the age-related increase of adipose tissue mass and partially improves the obese and diabetic phenotypes in mice (McPherron and Lee 2002). In human and mouse models, the higher levels of MSTN in muscle have been found to be positively related to obesity and type 1 and type 2 diabetes (Milan et al. 2004). Previously, researchers discovered that plasma and muscle MSTN protein levels increased with both body mass and the severity of insulin resistance in extremely obese and lean human subjects (Hittel et al. 2009). In myoblasts, MSTN mediated myostatin signalling by specifically inducing smad3 phosphorylation and interfering with the activity and expression of the myoblast differentiation factor MyoD, hence the inhibiting of the differentiation of myoblasts into myotubes (Langley et al. 2002). *In vitro*, recombinant MSTN predominantly promoted the proliferation of 3T3-L1 preadipocytes and reduced lipid accumulation in 3T3-L1 cells consequently by inhibiting the expression of critical lipogenic enzymes and promoting lipolytic enzyme expression (Langley et al. 2002).

Fibronectin type III domain-containing 5 (FNDC5), a well-defined myokine and also identified as an adipokine, has a critical role in the modulation of metabolism and protection against obesity. These important functions are mediated by irisin, a secretory peptide produced from proteolytic processing of FNDC5 (Rabiee et al. 2020). It was found that MSTN may be the upstream regulatory molecule of FNDC5 (Ge et al. 2017). However, whether MSTN is involved in weight loss caused by Rb1 has not been reported.

## Materials and methods

### Animals

We fed forty C57BL/6 male mice (six weeks old) a high-fat diet (HFD) for 12 weeks. Then these mice were divided into two groups when body weight reached approximately 55 g: the control (CTR) group (intraperitoneally (ip) administered with saline) and the Rb1 group (ip administered with Rb1, 40 mg/kg/d). Body weight was monitored every day. Mice fed a normal diet were randomly divided into 2 subgroups: the chow group (fed a normal diet and treated with saline) and chow + Rb1 group (fed a normal diet and treated with Rb1). Body weight was measured weekly. Mice were housed on a 12 h light/dark cycle. All animal protocols were approved by the Institutional Animal Care and Use Committee of Cheeloo College of Medicine, Shandong University (animal ethics number is KYLL-2018 (KJ) A-0072).

### RNA-Seq library generation and analysis

Total RNA was extracted using an RNeasy mini kit (Qiagen, Germany). With the guidance of the TruSeq™ RNA sample preparation guide, paired-end libraries were synthesized using the TruSeq™ RNA Sample Preparation kit (Illumina, USA). The products were then purified and concentrated by PCR to produce the final cDNA library, which was then quantified by Qubit® 2.0 Fluorometer (Life Technologies, USA) and verified by an Agilent 2100 bioanalyzer (Agilent Technologies, USA) to calculate the mole concentration and confirm the insert size. With the library diluted to 10 pM, a cluster was generated by cBot and then sequenced on an Illumina NovaSeq 6000 (illumina, USA). The differential expression of genes was identified with both a *p* value <0.05 and a fold-change of >1.5 between the two groups.

### Serum measurements

Serum total cholesterol (TC), TG, low density lipoprotein-cholesterol (LDL), high density lipoprotein-cholesterol (HDL), serum creatinine (Scr) and glutamic-oxalacetic transaminase were detected by assay kits (Changchun Huili Biotechnology Co., Ltd.).

### Metabolic cage

The O_2_ consumption (VO_2_), CO_2_ production (VCO_2_) and heat were detected by using an Oxymax/CLAMS animal metabolic system (Coulumbus Instruments). Experiments involving CTR and Rb1 treated mice were 20–22 weeks old. Mice were monitored for 48 h individually and data were collected every 30 min after 24 h of adaptation.

### Intraperitoneal glucose tolerance test

Four weeks after the application of Rb1, an intraperitoneal glucose tolerance test (IPGTT) was conducted. Mice were fasted overnight in advance and then intraperitoneally injected with glucose at a dose of 2 g/kg body weight. Glucose concentrations in blood taken from the tail vein of the mice were measured respectively at 0 (fasting), 15, 30, 60, and 120 min after glucose administration.

### C2C12 cell culture and treatment

C2C12 murine myoblasts were purchased from American Type Culture Collection (Manassas, VA, USA) and were cultured in Dulbecco’s modified Eagle’s medium (DMEM) with 10% foetal bovine serum (FBS), 100 μg/mL streptomycin, and 100 IU/mL penicillin. Differentiation medium was made up of DMEM containing 2% horse serum. When cell confluence reached 90%, the medium was changed to differentiation medium to induce differentiation of C2C12 cells. Skeletal muscle differentiation from C2C12 cells was induced in differentiation medium for 5–8 days (Baek et al. 2019). Then Rb1 (0, 10, 20, 40 μM) was added to the differentiated cells. After stimulation for 24 h, the cells were harvested.

#### 3T3-L1 cell culture and treatment

Mouse 3T3-L1 preadipocytes were purchased from ATCC and were cultured in high glucose DMEM containing 10% FBS in a saturated humidity atmosphere of 5% CO_2_ at 37 °C. The 3T3-L1 preadipocyte differentiation was assessed using the classic cocktail method. Briefly, cells were treated with a mixture of 1 μM dexamethasone, 10 μg/mL insulin and 0.5 mM 3-isobutyl-1-methylxanthine in DMEM for 2 days. Then, the medium was replaced by DMEM containing 10% FBS and 10 μg/mL insulin. After that, the cells were cultured for 2 more days, and the growth medium used for an additional day was composed of DMEM supplemented with 10% FBS. When lipid droplets accumulated in the cytoplasm in more than 90% of the 3T3-L1 cells, the cells could be further studied. The doses of Rb1 were 0, 20, and 40 μM (Guo et al. 2020). After stimulation for 24 h, the cells were harvested.

For cells with MSTN overexpression, 3T3-L1 induced adipocytes were divided into four groups: CTR + vehicle (C + V), Rb1 + vehicle (R + V), Rb1 + MSTN (Gene ID:17700, OBiO Scientific service, China) (R + M), and MSTN overexpression group (M). In the C + V group, control adenovirus (ADV) was added to cells. In the R + V group, 40 μM Rb1 and CTR ADV were added to cells. In the R + M group, 40 μM Rb1 and MSTN-ADV were added to cells. In M group, MSTN-ADV were added to cells. Rb1 was added to cells 24 h after viral transfection. The multiplicity of infection (MOI) was 50. After 24 h, the cells were harvested.

### Oil red O staining

Oil red O staining was used to detect lipid droplets in the cytoplasm of 3T3-L1 induced adipocytes. Cells were washed with PBS two times and fixed with 4% paraformaldehyde for 15 min at room temperature. Then, the cells were washed with PBS three times and 0.5% oil red O was added to the cells and incubated for 1 h. After three washes with PBS, the cells were observed under a microscope at 100× magnification. To quantify cellular lipids, stained cells were eluted with 100% isopropanol and incubated for 10 min. Then, 150 μL lysate was added to 96-well plates. Absorbance was determined at 500 nm with a full wavelength detector (SpectraMax M5/M5e, Molecular Devices).

### RT-PCR

The real-time PCR system was used to detect mRNA expression. Total RNA was extracted from C2C12 cells and 3T3-L1 cells by TRIzol reagent (Life Technologies). For reverse transcription (RT), a PrimeScript RT reagent Kit (Takara, Shiga, Japan) was used. As described previously, quantitative PCR was performed using SYBR Premix Ex Taq (Takara) and a Roche Light Cycler 480 II instrument in a 96-well plate following the manufacturer’s protocol. The primers below were used to amplify MSTN, FNDC5 and Actin. MSTN-F: TCACGCTACCACGGAAACAA; MSTN-R: AGGAGTCTTGACGGGTCTGA. FNDC5-F: TCATGTGGGCAGGTGTTATAG; FNDC5-R: TGTTATTGGGCTCGTTGTCCT. Actin-F: CCACACCCGCCACCAGTTCG, Actin-R: TACAGCCCGGGGAGCATCGT.

### Western blotting

RIPA lysis buffer supplemented with a complete protease inhibitor cocktail was used to lyse cells and tissues on ice. Equal amounts of protein (10 µg) were separated on a 10% SDS-PAGE gel. Then, the proteins were was transferred to PVDF membranes (0.22 µm, Millipore). Membranes were blocked in 5% skim milk and incubated with primary antibodies against MSTN (AF788; R&D Systems), FNDC5 (ab131390; Abcam), MYH4 (20140-1-AP, Proteintech) and Tubulin (11224-1-AP, Proteintech) was used as CTR.

### Histopathological staining

The inguinal white adipose tissue (ingWAT), epididymis white adipose tissue (epiWAT), brown adipose tissue (BAT) and skeletal muscle were taken immediately after mouse sacrifice. One half of the tissues were fixed in 4% formaldehyde overnight. The other half of the tissues were frozen at −80 °C for molecular experiments. Tissues embedded in paraffin were cut into 5 μm sections. After they were dewaxed, ingWAT, epiWAT and BAT sections were stained with haematoxylin and eosin (H&E). The size of adipocytes was determined by measuring of cell diameters. Immunohistochemical staining was performed with MSTN antibody (AF788; R&D Systems; 1:100). The secondary antibody was HRP-conjugated rabbit anti-goat IgG (H + L) (SA00001-4, Proteintech;1:200). Livers were frozen in OCT embedding medium and then stained with Oil Red O. The sections were photographed by a whole slide imaging scanner (Pannoramic SCAN II, 3 D HISTECH).

### ELISA

The MSTN in serum, culture supernatant of differentiated C2C12 cells and 3T3-L1 cells were determined via ELISA kits (MSTN, TAE-626 m, Tianjin Anoric Biotechnology Co., Ltd) according to the manufacturer's protocol. Plates were read on a spectrometer at 450 nm wavelength after the procedure. The results were converted to numeric values by using standard curves.

### Statistical analysis

SPSS 19.0 (SPSS Inc., Chicago, IL) was used to statistical analysis and graphpad 5.0 was used to draw statistical figures in this article. All results are represented as the mean ± SEM. Student’s *t*-test was used to compare two groups and one-way ANOVA with Tukey’s *post hoc* test was used to compare multiple groups. *p* < 0.05 was considered statistically significant.

## Results

### Rb1 decreased body weight and cholesterol and triglyceride levels in obese mice

**Figure 1.**
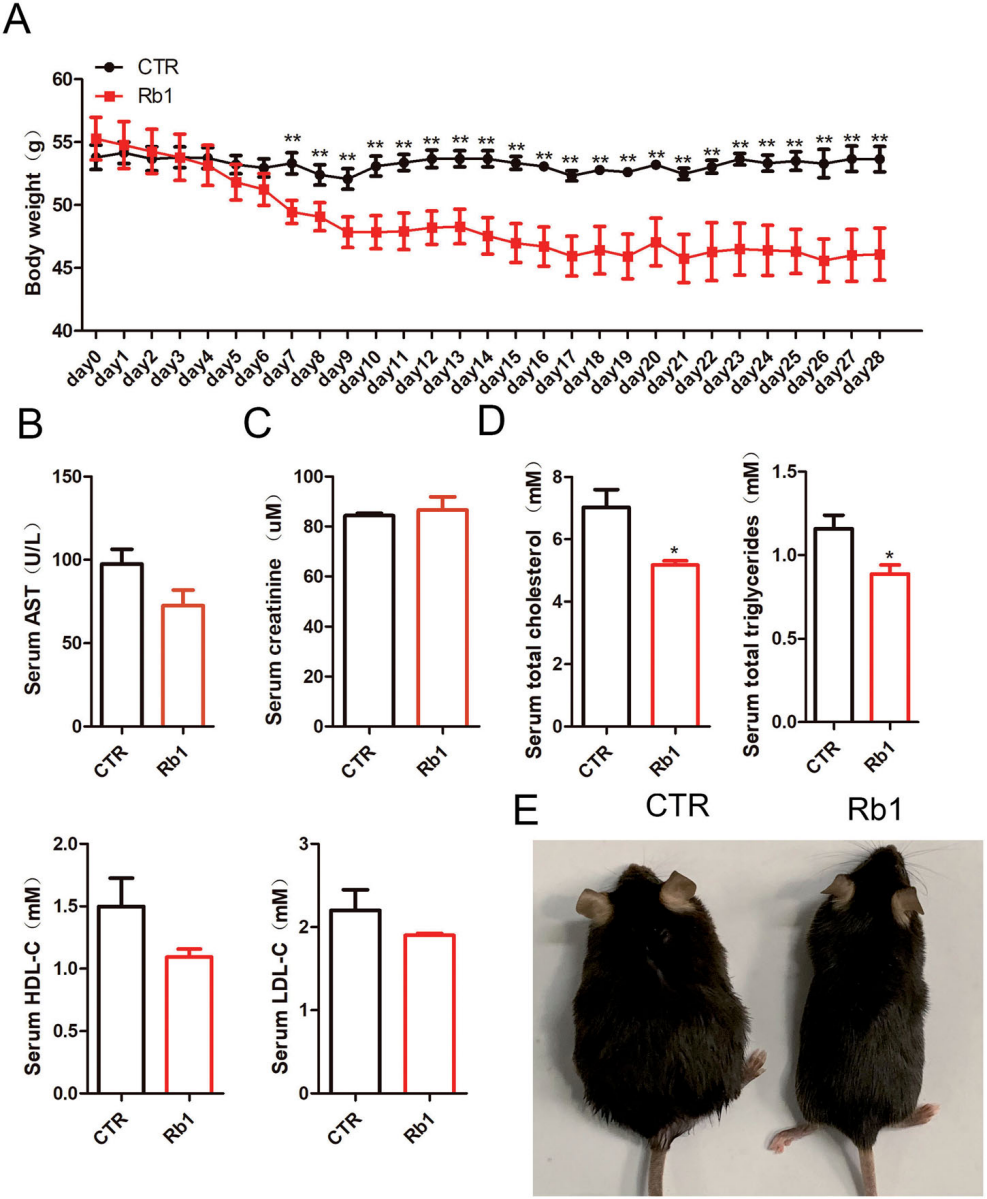
Effect of Rb1 on body weight and cholesterol and triglyceride levels in obese mice. (A). Variations in mouse body weight between the two groups. (B). Serum levels of aspertate aminotransferase (AST), (C) creatinine (Cr), (D) Total cholesterol (TC), triglycerides (TG), high-density lipoprotein cholesterol (HDL-C) and low density lipoprotein cholesterin (LDL-C). (E) Representative mouse photographs in the two groups. *p < 0.05 vs. CTR group, **p < 0.01 vs. CTR group (*n* = 5).

To investigate how Rb1 affects body weight in obese mice, a mouse model of obesity was established through HFD feeding. After HFD feeding for 12 weeks, the body weight of the mice was dramatically increased. We divided the mice into two groups: the CTR group and the Rb1 group. After Rb1 injection, the body weight was measured every day. The results showed that the body weight of obese mice was predominantly reduced by Rb1 treatment from 7 days after Rb1 injection (Figure 1(A)). But there was no alteration of body weight between normal control mice and Rb1 + normal control mice (Supplemental Figure 1(A)). Liver function and kidney function were not affected by Rb1 injection (Figure 1(B,C)). Serum total cholesterol and total triglycerides showed decreased expression in Rb1 treated mice. There were no significant differences in the CTR group and Rb1 group in terms of high -density lipoprotein cholesterol and low density lipoprotein cholesterol (Figure 1(D)). Mice treated with Rb1 became significantly thinner (Figure 1(E)).

### Rb1 improved glucose tolerance and increased basic metabolic activity in obese mice

**Figure 2.**
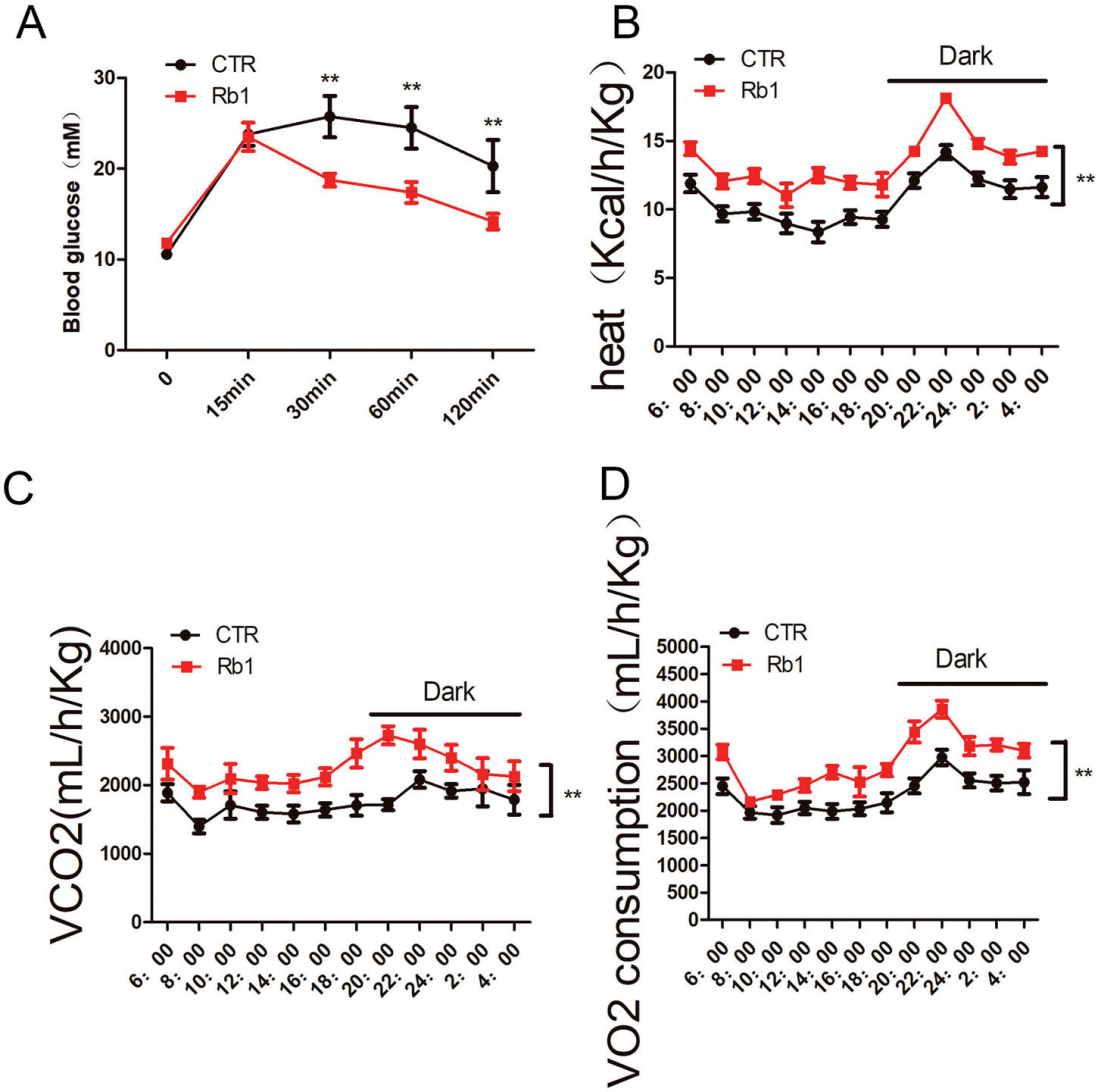
Effect of Rb1 on glucose tolerance and metabolism. (A) Glucose tolerance test. (B) Heat production. (C) Carbon dioxide production. (D) Oxygen consumption. **p < 0.01 vs. CTR group (*n* = 5).

Obesity is tightly connected with insulin resistance and glucose intolerance. Compared with HFD-fed CTR mice, Rb1 group mice showed improved glucose intolerance (Figure 2(A)).

We also evaluated the effect of Rb1 on basic metabolic activity. Under the conditions of HFD feeding, mice in the Rb1 group showed increased oxygen consumption, carbon dioxide production and heat production (Figure 2(B–D)). The increased resting metabolic rate might explain the decreased body weight seen in mice injected with Rb1.

### Rb1 improved adipocyte hypertrophy and fatty liver in obese mice

**Figure 3.**
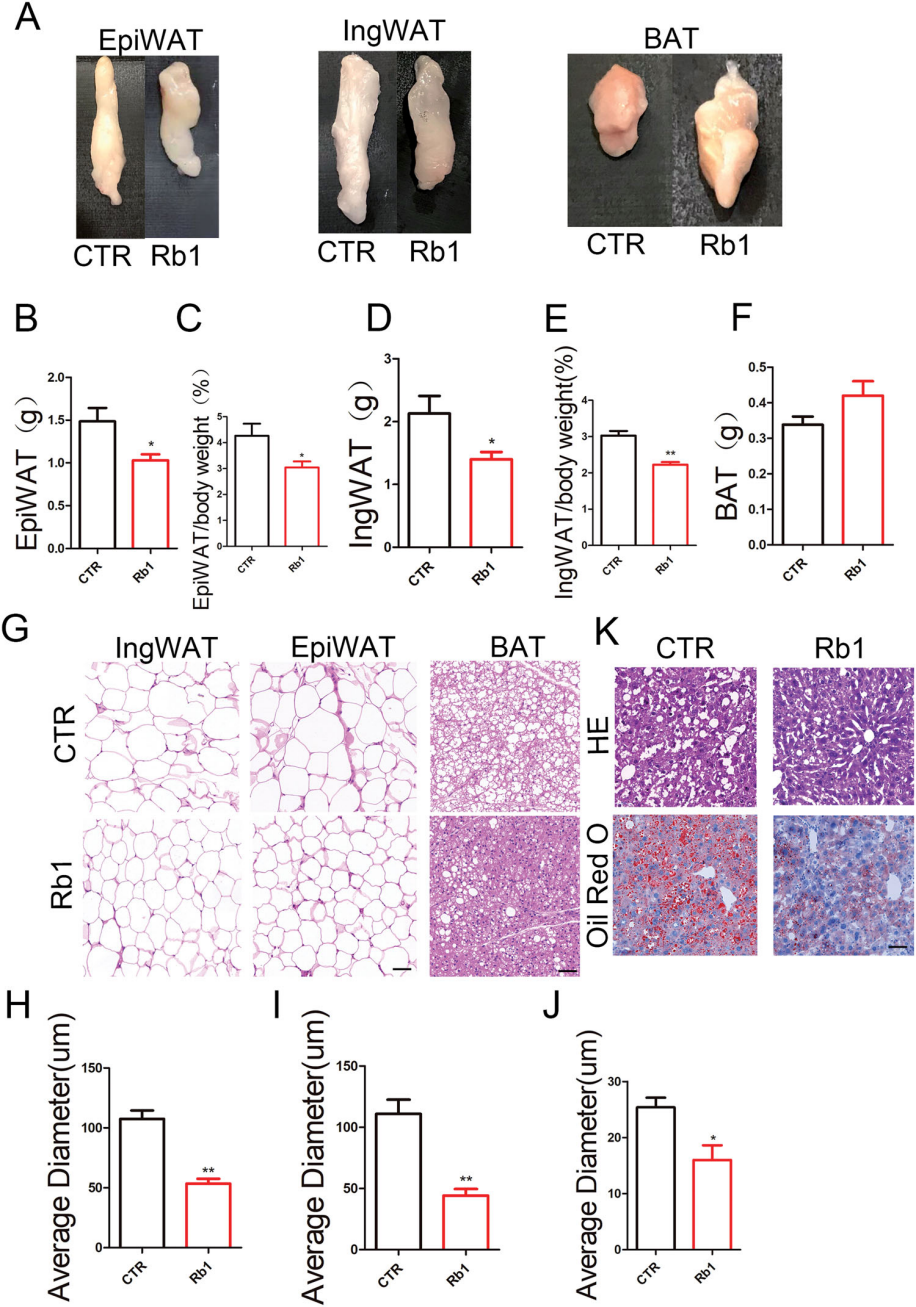
Effect of Rb1 on adipocyte hypertrophy and fatty liver. (A) Representative photographs of epididymis white fat (epiWAT), inguinal white fat (ingWAT) and BAT. (B) Quantification of epiWAT. (C) Quantification of epiWAT/body weight. (D) Quantification of ingWAT. (E) Quantification of ingWAT/body weight. (F) Quantification of BAT. (G) Representative HE images of epiWAT, ingWAT and BAT in the two group of mice. (H) Quantification of average ingWAT diameters. (I) Quantification of average epiWAT diameters. Scale bar = 100 μm. (J) Quantification of average BAT diameters. Scale bar = 50 μm. (*n* = 5). (K) Representative HE staining and oil red O staining in liver. Scale bar = 50 μm. (*n* = 4). *p < 0.05 vs. CTR group;**p < 0.01 vs. CTR group.

A decrease in adipose tissue mass can be ascribed to a decrease in adipocyte number or size on account of abnormal differentiation, or both. To reveal the mechanism of decreased adiposity in Rb1 treated mice, we measured the adipocyte size and weight in the adipose tissue of CTR and Rb1 treated mice. There were decreased weights of epiWAT and ingWAT in Rb1-treated mice (Figure 3(A–F)). H&E staining indicated smaller adipocytes in both ingWAT, epiWAT and BAT of Rb1 treated mice than CTR mice (Figure 3(G–J). Because of the close association between hepatic steatosis and obesity, we assessed the effect of Rb1 on hepatic lipid deposition into assessment. The results showed that fatty liver was alleviated in Rb1-treated mice (Figure 3(K)).

### Rb1 decreased MSTN mRNA and protein expression in adipose tissue, skeletal muscle and serum

**Figure 4.**
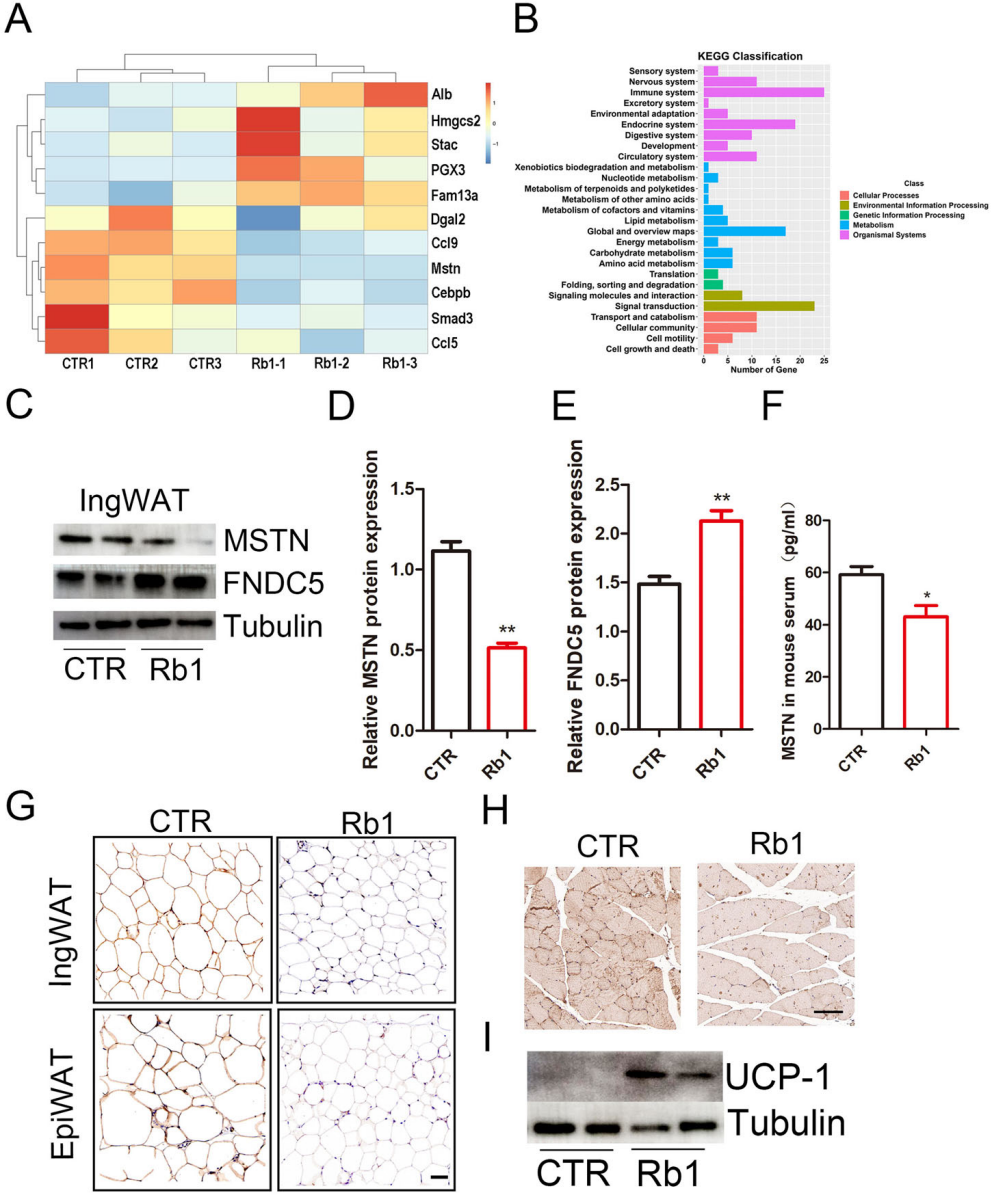
Effect of Rb1 on MSTN expression in adipose tissue. (A) Representative genes with >1.5-fold upregulation or >1.5-fold downregulation in CTR and Rb1 mouse adipose tissue (*n* = 3). (B) The signalling pathways of Rb1 regulate the genes involved in. (C) Protein expression of Myostatin (MSTN) and Fibronectin type III domain-containing 5 (FNDC5) in adipose tissue (*n* = 3). (D) Quantification of relative MSTN protein expression. (E) Quantification of relative FNDC5 protein expression. (F) The MSTN in mouse serum (*n* = 5). (G) Immunohistochemical staining showed the expression of MSTN in epiWAT and ingWAT (*n* = 5). (H) Representative photograph of MSTN expression in CTR and Rb1 mouse skeletal muscle. (I) Western blot analysis of uncoupling protein 1 (UCP-1) expression in white adipose tissue. *p < 0.05 vs. CTR group; **p < 0.01 vs. CTR group.

To explore the specific mechanism of how Rb1 caused the reduction of adipocyte hypertrophy, we used a gene array assay of adipocyte tissues from CTR and Rb1 treated mice. In Rb1 treated mice, the expression of adipogenic transcription factors such as CCAAT/enhancer-binding protein β (C/EBPβ) was decreased. The MSTN was dramatically decreased in adipose tissue in Rb1 treated mice (Figure 4(A)). The results also showed that Rb1 is involved in many biological processes, such as lipid metabolism and energy metabolism, etc. (Figure 4(B)). We further detected the protein level of MSTN in ingWAT, and consistent with the results found at the mRNA level, MSTN protein level was decreased in Rb1 group (Figure 4(C,D)). The MSTN in serum was also detected by ELISA, results showed that there was decreased MSTN in Rb1 treated mouse serum (Figure 4(F)). The importance of MSTN in obese mice has been deeply studied, and Rb1 may improve obese mice through MSTN. As a molecule downstream of MSTN, FNDC5 also plays an important role in obesity and diabetes. We found a higher expression of FNDC5 expression in adipocyte tissue (Figure 4(C,E)). Immunohistochemical staining of adipose tissue and skeletal muscle showed that there was decreased expression of MSTN in Rb1 treated mice (Figure 4(G,H)). BAT is characterized by the expression of uncoupling protein 1 (UCP1), and the expression level of UCP1 represents the volume of BAT. Our results showed that Rb1 promoted UCP1 expression in white adipocyte tissue, thus promoting browning of white fat (Figure 4(I)).

### Rb1 decreased MSTN mRNA and protein expression in differentiated C2C12 myoblasts and 3T3-L1 adipocytes

**Figure 5.**
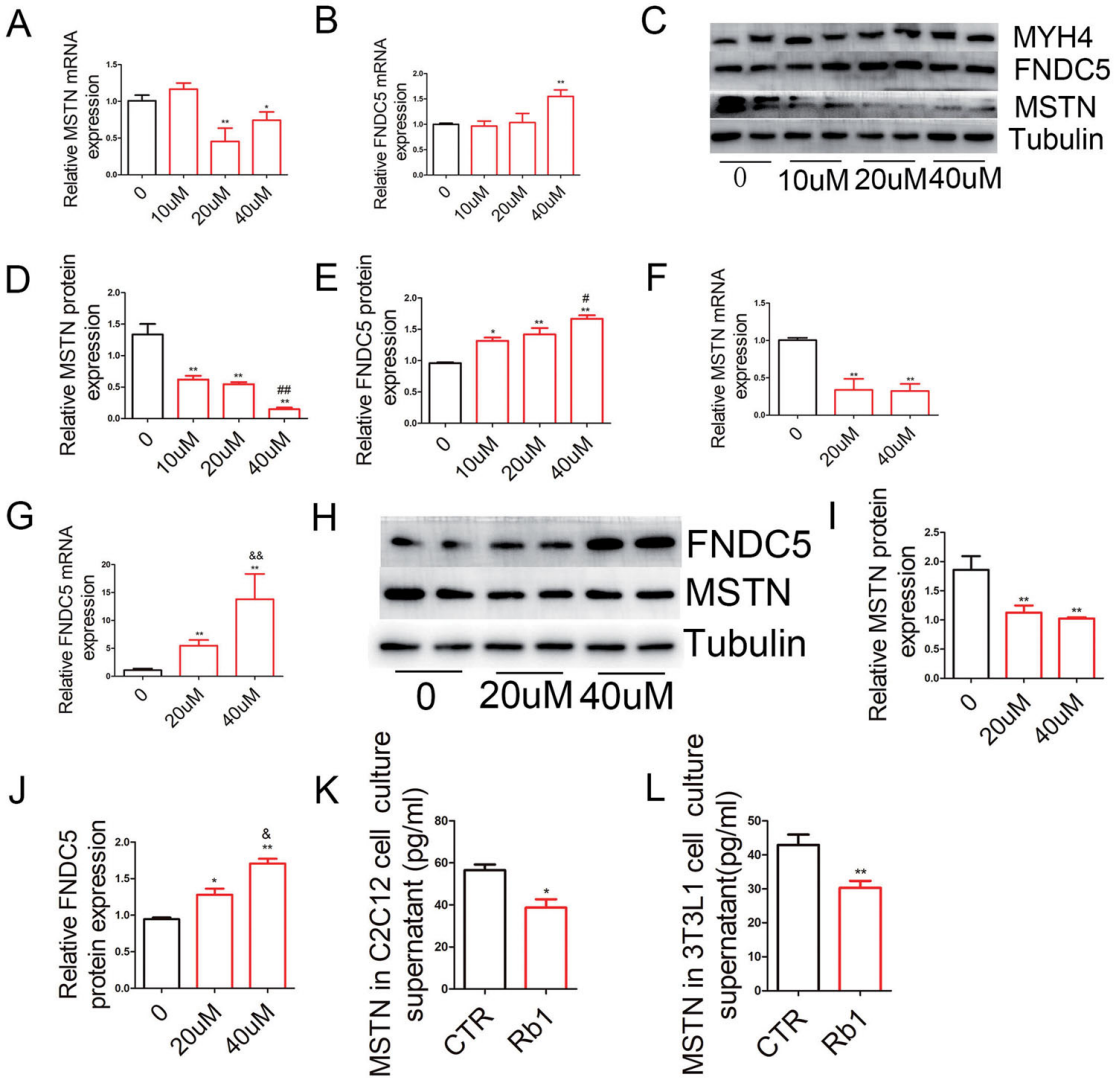
Rb1 regulates MSTN and FNDC5 mRNA and protein expression in differentiated C2C12 myoblasts and 3T3-L1 adipocytes. (A-B). The effect of 0, 10, 20, 40 μM Rb1 on MSTN and FNDC5 mRNA expression in differentiated C2C12 myoblasts. (C) Western blot analysis of MYH4, MSTN, and FNDC5 expression in differentiated C2C12 myoblasts. (D-E). Statistical analysis of MSTN and FNDC5 protein levels. (F-G) The effect of 0, 20, and 40 μM Rb1 on MSTN and FNDC5 mRNA expression in differentiated 3T3-L1 adipocytes. (H) Western blot analysis of MSTN and FNDC5 expression in differentiated 3T3-L1 adipocytes. (I-J) Statistical analysis of MSTN and FNDC5 protein levels. (K) The MSTN expression in the culture supernatant of differentiated C2C12 myoblasts. (L) The MSTN expression in the culture supernatant of differentiated 3T3-L1 adipocytes (*n* = 5). *p < 0.05 vs. CTR group, **p < 0.01 vs. CTR group, # *p* < 0.05 vs. 10 μM Rb1, ##*p* < 0.01 vs. 10 μM Rb1, & *p* < 0.05 vs. 20 μM Rb1, &&*p* < 0.01 vs. 20 μM Rb1.

To further study the effect of Rb1 on obesity, we cultured C2C12 cells and 3T3-L1 cells *in vitro* with and without Rb1 treatment respectively. Differentiated C2C12 myoblasts expressed high levels of myosin heavy chain 4 (MYH4) (Brearley et al. 2021), we found a high expression of MYH4 in C2C12 cells induced myoblasts (Figure 5(C)). Differentiated 3T3-L1 adipocytes and C2C12 cells were treated with Rb1 at different concentrations for 24 h. The results showed that the MSTN mRNA was decreased in cells treated with 20–40 μM Rb1 and that FNDC5 mRNA was increased in cells treated with 40 μM Rb1 in differentiated C2C12 cells (Figure 5(A,B)). The FNDC5 protein level was upregulated when differentiated C2C12 cells were treated with 10–40 μM Rb1, and the MSTN protein level was downregulated by treatment with Rb1 from 10 to 40 μM Rb1 (Figure 5(C–E)).

In differentiated 3T3-L1 adipocytes, 20 and 40 μM Rb1 decreased MSTN mRNA expression and 40 μM Rb1 increased FNDC5 mRNA expression (Figure 5(F,G)). At the protein level, 20 μM and 40 μM Rb1 significantly increased FNDC5 expression and decreased MSTN expression (Figure 5(H–J)).

We also detected the MSTN protein levels in C2C12 cells and 3T3-L1 cell culture supernatants. Our results showed that there was decreased MSTN in Rb1 treated cells (Figure 5(K,L)).

### Rb1 inhibited lipid deposition through MSTN in 3T3-L1 induced adipocytes

**Figure 6.**
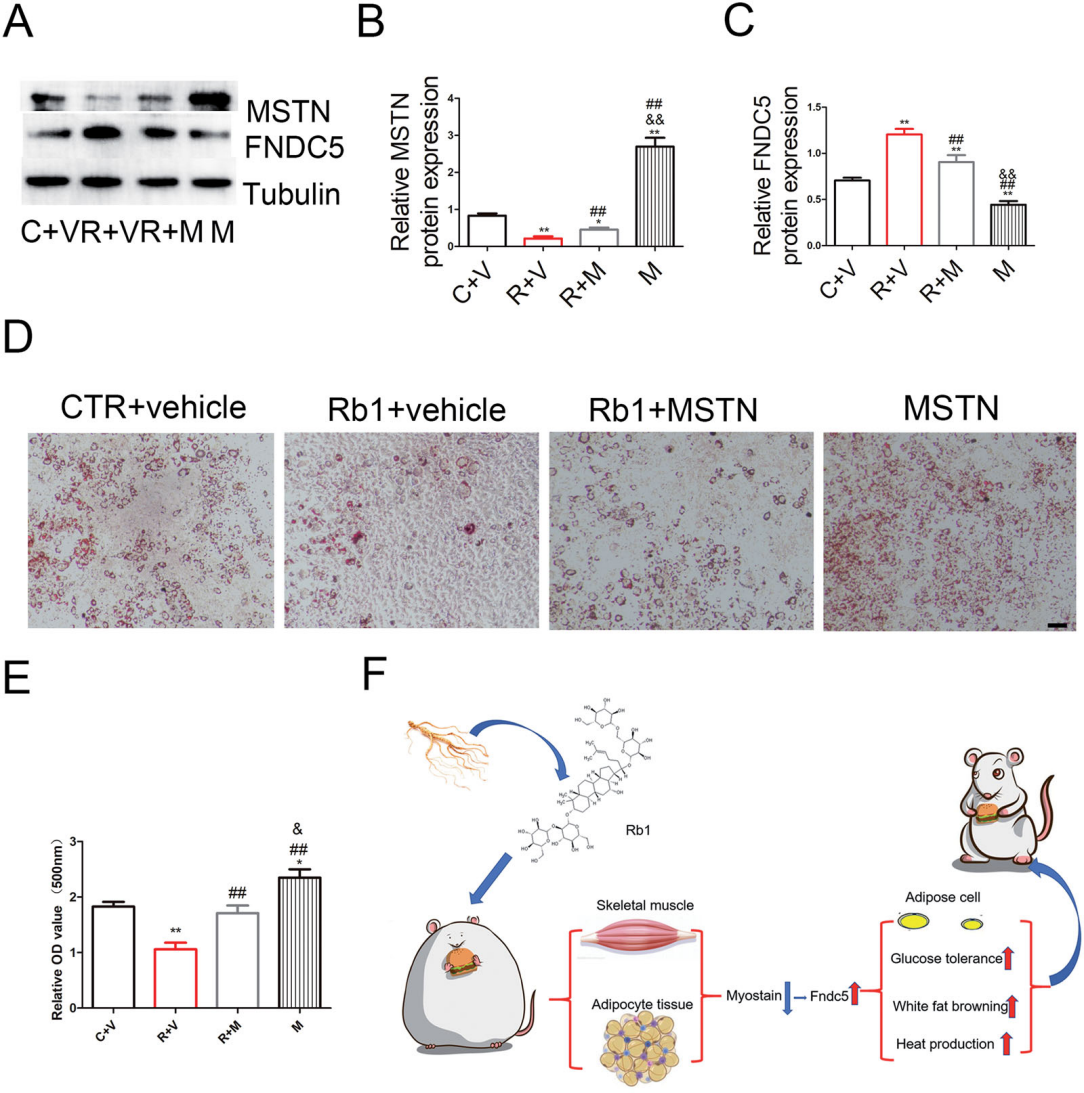
Rb1 inhibit lipid deposit through MSTN in 3T3-L1 induced adipocytes. (A) WB analysis of MSTN and FNDC5 expression. (B-C) Statistical analysis of A. (D) Representative Oil red O staining. (E). Statistical analysis of D. Scale bar = 50 μm. (F) Schematic diagram about the mechanism of Rb1 in adipose tissue and obesity (*n* = 5). **p < 0.01 vs. CTR + vehicle (C + V) group, #*p* < 0.05 vs. Rb1 + vehicle (R + V) group, ##*p* < 0.01 vs. R + V group, &&*p* < 0.01 vs. Rb1 + MSTN (R + M) group.

To further study the mechanism by which Rb1 regulates lipid deposition, MSTN was overexpressed in 3T3-L1 induced adipocytes. WB showed that Rb1 decreased MSTN expression and increased FNDC5 expression. However, MSTN overexpression counteracted the effect of Rb1 (Figure 6(A–C)). Oil red O staining showed that Rb1 decreased lipidosis but this effect was offset by the overexpression of MSTN (Figure 6(D–E)).

## Discussion

In this research, we explored the role of Rb1 in obesity and metabolic disturbances in mice. The results showed that Rb1 treatment reduced body weight and adipocyte enlargement and increased the resting metabolic rate in HFD-induced obese C57BL/6 mice. Rb1 treatment decreased MSTN in adipose tissue, skeletal muscle and serum *in vivo*. *In vitro*, Rb1 treatment decreased MSTN mRNA and protein expression in differentiated C2C12 cells and 3T3-L1 cells. We also found that Rb1 treatment promoted FNDC5 mRNA and protein expression. MSTN overexpression counteracted the effect of Rb1 in inhibiting lipid deposition in 3T3-L1 cells. These results indicated that the MSTN/FNDC5 signalling pathway was involved in Rb1 ameliorating obesity status.

Under the influence of environmental and genetic factors, obesity is associated with energy-balance dysregulation (Kopelman 2000). The biological characteristics of obesity are adipocyte hypertrophy and excessive adipose accumulation. The maintenance of lipid homeostasis depends on lipolysis and lipogenesis in adipocytes. Adipose tissue, as an important energy storage organ, has become a therapeutic target for obesity. BAT plays an important role in promoting total energy consumption in regulating the body's energy metabolism by producing heat (Algire et al. 2013). It is more interesting that brown adipose tissue has the ability to protect against obesity by releasing batokines, clearing triglycerides, and reducing insulin resistance (Jeremic et al. 2017). Browning of white fat helps to restrict obesity and obesity-related disorders. Although the most common factor for fat browning has been exercise, there may be other factors, such as Rb1, that are involved. In our study, we found a smaller adipocyte diameter and increased basic metabolic activity in Rb1 treated mice.

Ginseng, as the root of the *Panax ginseng*, has been believed to prolong life and resist ageing. Ginsenosides are the most important bioactive components of ginseng. Studies have shown that there are multiple benefits of ginsenosides in the circulatory, immune, endocrine and central nervous systems (Kim 2018). Rb1, as the richest and most representative ginsenoside, has been reported to influence both obesity and diabetes. For example, Xiong et al. (2010) found that Rb1 significantly reduced food intake, weight gain, and body fat content, increased energy expenditure and improved glucose tolerance in HFD-induced obese mice. Guo et al. (2020) found that Rb1 could markedly diminish the body weights of mice with HFD-induced obesity and the mechanism involved was that Rb1 enhanced AQP7 expression both *in vivo* and *in vitro*. AQP7 participates in the process of the transport of triglycerides and adipogenesis in adipocytes (Hibuse et al. 2006). The expression level of AQP7 is closely associated with the occurrence of obesity and type 2 diabetes (Prudente et al. 2007). In Xiong et al. (2010) study, they found that Rb1 suppressed food intake, body weight gain and body fat content and increased energy expenditure by stimulating c-Fos expression in brain areas and activating the PI3K/Akt signalling pathway and inhibiting NPY gene expression in the hypothalamus. In Lim et al. (2019) study, they found that Rb1 promoted browning of white fat through β-3 adrenergic receptor activation and increased the expression of UCP-1. In Shen et al. (2013) study, they found that Rb1 ameliorated fatty liver by activating AMP-activated protein kinase in obese rats.

In our study, we found that Rb1 decreased body weight, cholesterol, triglycerides and body fat content in obese mice. Oil red O staining showed that the lipids in the liver in Rb1- treated mice were significantly decreased. Rb1 improved glucose tolerance and increased basic metabolic activity. Our results are consistent with those of published studies. Changes in food intake in mice treated with Rb1 are still controversial. Some studies have found that Rb1 reduces food intake (Xiong et al. 2010), while others have found that Rb1 does not affect food intake (Shin and Yoon 2018). In our study, we found that there was a decrease in food intake in Rb1 treated mice, but the difference was not statistically significant (Supplemental Figure 1B). Some studies have found that Rb1 promotes adiogenesis. For example, in Shang et al. (2007) study, they found that Rb1 promoted adiogenesis in 3T3-L1 cells, and the mechanism involved the enhanced expression of PPARgamma and C/EBPalpha. Chan et al. (2012) found that Rb1-promoted adipogenesis via PPARγ binding and miR-27b regulation. In the above studies, Rb1 was used to stimulate 3T3-L1 cells, then the cells were induced to transform into adipocytes. In this process, Rb1 promoted adipogenesis in 3T3-L1 cells. However, in our study, we first induced 3T3-L1 cells to become adipocytes, and then stimulated the cells with Rb1 to observe the effect, and finally found that Rb1 can reduce lipid deposition in adipocytes. In Lim et al. (2019) study, they also found that Rb1 induced lipolysis in 3T3-L1 adipocytes. However, some studies have also reported that ginseng extract can inhibit lipodysis. Wang et al. (2006) found that ginseng extract inhibited lipolysis by activating PDE4 in rat adipocytes. In Yu et al. (2015) study, they found that Rb1 at a concentration of 10 μM can inhibit lipolysis in 3T3-L1 adipocytes. In our study, we found that a concentration of 40 μM Rb1 significantly decreased lipidosis in 3T3-L1 adipocytes. The difference in our studies may be due to the dose of Rb1 applied.

Using a gene array assay of the control and Rb1 treatment mouse adipose tissues, we found that the expression of MSTN was significantly decreased. This is a new finding that has not been reported in previous studies. Further study also found decreased MSTN protein levels in skeletal muscle, adipose tissue and serum. Overexpression of MSTN in Rb1 treated 3T3-L1 induced adipocytes counteracted the effect of Rb1 in inhibiting lipidosis (Figure 6(A–E)). The traditional view is that MSTN only acts as a negative factor for skeletal muscle growth and plays a vital role in inhibiting skeletal muscle growth (McPherron and Lee 1997). Recent studies have shown that MSTN is expressed not only in skeletal muscle but also widely in mammalian adipose tissue, breast, heart tissue, lymphatic tissue and other tissues (Lyons et al. 2010). In 3T3-L1 preadipocytes, MSTN mainly inhibits fat production. In the process of differentiation, 3T3-L1 preadipocytes treated with MSTN significantly inhibited adipogenesis by regulating PPARγ and C/EBP-β (Takahata et al. 2004). Paradoxically, a decrease or knockout of MSTN leads to suppression of body fat accumulation and an increase in myogenesis (Lin et al. 2002; McPherron and Lee 2002). Researchers have found that MSTN-knockout (MSTN^−/−^) mice exhibit browning of white adipose tissues. The mechanism involved was that PGC1α and FNDC5 were activated in MSTN^-/-^ skeletal muscle in mice (Shan et al. 2013). Ge et al. (2017) also found that MSTN - treated myoblasts inhibited FNDC5 expression, but MSTN inhibition increased FNDC5 levels in the circulation and muscles. MSTN^−/−^ adipocytes showed enhanced FNDC5 expression. MSTN^−/−^ adipocytes have improved mitochondrial function and increased mitochondria and heat production (Kong et al. 2018). Thus, FNDC5 plays an important role in MSTN regulation of weight loss in mice. Studies have shown that FNDC5 overexpression enhances energy expenditure, insulin sensitivity and lipolysis and decreases hyperglycaemia, hyperlipidaemia, hyperinsulinemia, norepinephrine levels and blood pressure in obese mice (Xiong et al. 2015). In our study, we also found increased expression of FNDC5 in skeletal muscle, adipose tissue, differentiated C2C12 cells and 3T3-L1 cells.

To summarize, we demonstrate that Rb1 is a negative regulator of obesity. Mice treated with Rb1 inhibited HFD-induced obesity and improved glucose intolerance and fat liver and adipose function. MSTN and FNDC5 were involved in this process. It is a potential therapeutic that Rb1 may be for the treatment of obesity and obesity-related metabolic disorders (Figure 6(F)).

## Conclusions

Our results showed that Rb1 may ameliorate obesity in part through the MSTN/FNDC5 signalling pathway. Our study provides important experimental evidence for the treatment of obesity by Rb1. Rb1 can be used as an effective drug in the treatment of human obesity.

## Supplementary Material

Supplemental Material

Supplemental Material

## Data Availability

The datasets used or analyzed during the current study are available from the corresponding author on reasonable request.
