# Supplementary figures and images for: Myostain is involved in ginsenoside Rb1-mediated anti-obesity
Source: Pharm Biol. 2022 May 31;60(1):1106–15. doi: 10.1080/13880209.2022.2074056 (PMC9176416; doi:10.1080/13880209.2022.2074056)

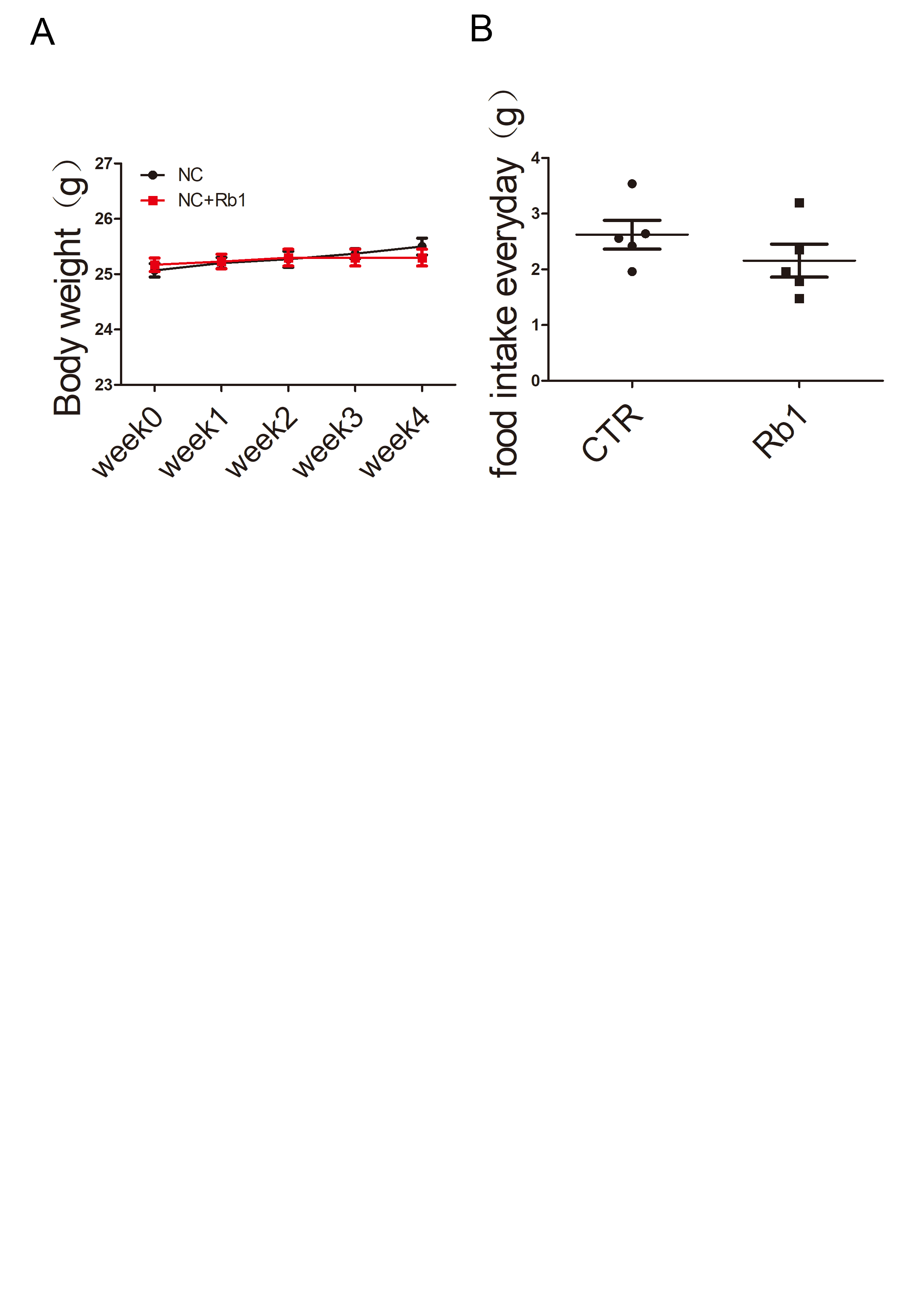

Supplement: Supplemental Material [file IPHB_A_2074056_SM0803.tif]
