# Supplementary material for: Myostain is involved in ginsenoside Rb1-mediated anti-obesity
Source: Pharm Biol. 2022 May 31;60(1):1106–15. doi: 10.1080/13880209.2022.2074056 (PMC9176416; doi:10.1080/13880209.2022.2074056)
Supplement: Supplemental Material [file IPHB_A_2074056_SM0805.docx]

**Supplemental Figure. 1 The body weight of normal diet mice and food intake of obese mice.** **A.** Variations of the mouse body weight from normal control (NC) group and NC + Rb1 group. (n=3). **B.** Food intake of obese mice. (n=5).
